# The COMPWALK-ACL: A Dataset of Multi-pace IMU Gait Kinematics in Adolescents, Adults, and ACL Injured Patients

**DOI:** 10.1038/s41597-025-06307-8

**Published:** 2025-12-09

**Authors:** Tomer Yona, Bezalel Peskin, Arielle Fischer

**Affiliations:** 1 Department of Biomedical Engineering, Technion, Israel Institute of Technology, Haifa, Israel; 2 Orthopedic Department, Rambam Health Care Campus, Haifa, Israel

**Keywords:** Outcomes research, Quality of life

## Abstract

Gait analysis provides objective, quantitative parameters essential for assessing mobility, identifying movement impairments, and monitoring the progress of rehabilitation. While traditional lab-based systems offer high accuracy, wearable Inertial Measurement Units (IMUs) enable portable, cost-effective gait assessments outside the laboratory environment. However, the reliability and applicability of IMU-derived data across diverse populations and walking conditions require robust datasets. This paper presents a lower limb kinematic dataset acquired with the Xsens Awinda IMU system. Data were collected from 92 unique participants: healthy adults (n = 25), healthy adolescents (n = 27), and individuals with ACL injuries assessed before surgery (n = 40), with 27 completing a follow-up three months post-reconstruction. Participants walked overground at self-selected slow, normal, and fast speeds. The dataset contains spatiotemporal parameters, as well as lower limb joint kinematics. It enables research on normative gait across age groups, the effects of ACL injury and early recovery on movement patterns, and the development of IMU-based gait analysis methods under different walking speeds and clinical conditions.

## Background & Summary

Human gait provides objective information about an individual’s movement capabilities, neurological function, and musculoskeletal health^1,2^. Consequently, gait parameters such as lower limb kinematics and kinetics, alongside spatiotemporal metrics, are recognized for their clinical utility in diagnosing conditions, planning personalized treatments, and monitoring recovery, such as following Anterior Cruciate Ligament (ACL) reconstruction^3–6^. Historically, gait analysis has required specialized laboratory settings with multiple optical motion capture cameras. While accurate, these systems are limited to controlled environments and are not suitable for use outside the laboratory^7^.

Wearable sensors, such as Inertial Measurement Units (IMUs), offer a portable and cost-effective alternative that enables gait assessment in real-world and clinical settings^8^. IMUs measure linear acceleration and angular velocity of body segments, which can be used to estimate their orientation and, consequently, joint kinematics.

Despite these advantages, broader clinical adoption of IMU-based gait analysis necessitates robust accuracy and reliability across diverse populations and walking conditions^9^. However, IMUs have mainly been focused on single tasks, often limited to jump-landing or hopping tasks, resulting in a scarcity of generalizable, standardized datasets suitable for analyzing walking in clinical populations^10^.

In particular, there is a need for normative IMU datasets spanning different age groups, such as adolescents and adults, as well as clinical populations, including ACL-injured participants, where gait patterns are altered^11,12^. ACL injuries are associated with altered knee flexion and extension angles, reduced symmetry, and compensatory hip or ankle strategies following surgery^6,13–15^. Additionally, variations in walking speed, which significantly influence gait patterns, should be considered in any comparative analysis^12,16^.

Yet, few publicly available datasets systematically incorporate variation in walking speed across both healthy and ACL-injured populations, despite the fact that speed strongly modulates joint kinematics and compensatory strategies^10,17^. Prior datasets often focus exclusively on healthy adults, emphasize environmental variability over clinical populations, or lack repeated measures across recovery stages. Additionally, most do not include pre- and post-operative measures within the same individuals^18–21^.

Compared to existing datasets, the COMPWALK-ACL dataset (COMParing multi-pace WALKing kinematics via IMU in healthy adolescents, adults, and individuals with ACL injury) provides a unique contribution. It is an IMU-based gait dataset comprising data from 92 participants: 25 healthy adults, 27 healthy adolescents, and 40 individuals with ACL injury, of whom 27 completed a follow-up assessment three months after ACL reconstruction. Each participant completed overground walking at three self-selected speeds: slow, normal, and fast. This design systematically integrates speed variation, age stratification, and pre- and post-operative measures within a single resource.

These limitations highlight the need for structured IMU-based reference datasets that facilitate robust interpretation and modeling of gait across various age groups, clinical conditions, and walking intensities. To address this gap, we developed the COMPWALK-ACL dataset as an openly available resource capturing both age-related variability and post-surgical changes using a standardized IMU protocol. This dataset supports within-subject and between-group analyses under ecologically valid conditions, facilitating the study of post-ACL biomechanical adaptations as well as the development of normative gait references for both youth and adults.

## Methods

This dataset comprises three cohorts that followed a similar gait protocol: 25 healthy adults (IRB approval number 143-2022), 27 healthy adolescents (IRB approval number 174-2023), and 40 adults with ruptured ACLs (IRB approval number 0089-21-RMB), of whom 27 completed a follow-up assessment at three months post-reconstruction.

Healthy adults were recruited through campus flyers, social media postings, and word of mouth. Adolescent participants were recruited through a national science program for gifted high school students. Participants with ACL injuries were recruited from patients scheduled for ACL reconstruction at the Orthopedic Center, Rambam Health Care Campus (Haifa, Israel).

All participants provided written informed consent for study participation and for future research use of their de-identified data (secondary-use clause). For adolescent participants, written consent was also obtained from their legal guardians. Refusal of the secondary-use clause did not affect eligibility for participation. Only participants who explicitly consented to the secondary use of their data were included in the publicly shared dataset.

To protect participant information, all data were de-identified at the point of collection. Each participant was assigned a unique, non-identifiable study code, and no personally identifying information is included in the final dataset.

### Participants

The healthy adults’ cohort included healthy males and females aged 18–45 years. Participants were excluded if they had any of the following: inability to provide informed consent or understand the study protocol, current pregnancy, known cardiovascular, neurological, or respiratory conditions, allergies to adhesive or silver materials, lower limb pain within the past six months, prior orthopedic surgery in the lower limbs, or a history of lower limb or back fractures, surgeries, known neuropathies, active cancer, or inflammatory arthritis.

The healthy adolescents’ cohort consisted of healthy males and females aged 15–17 years, with written informed consent provided by a parent or guardian. Exclusion criteria were inability to understand the study protocol, pregnancy, cardiovascular, neurological, or respiratory conditions, and any history of lower limb orthopedic pathologies or surgeries.

The ACL injury cohort included participants with ACL injuries, aged 18–40 years, who were scheduled for primary ACL reconstruction (ACLR) using hamstring, bone-patellar tendon-bone, or quadriceps tendon grafts. All ACLR surgeries were conducted by one of five senior orthopedic surgeons at a single orthopedic center. Participants were excluded if they had multi-ligament injuries, significant meniscal damage requiring modified weight-bearing protocols, previous knee surgeries or fractures in either leg, known neuropathies, active cancer, or inflammatory arthritis.

A summary of demographic characteristics for each cohort is presented in Table 1.

**Table 1:** Demographic details of the participants grouped by cohort.

Cohort	Healthy adults (n = 25)	Healthy adolescents (n = 27)	ACLD (n = 40)	ACLR (n = 27)
**Age (year)**	28.5 ± 5.7	15.7 ± 0.6	23.9 ± 5.9	23.6 ± 5.8
**Sex, n (%)**				
Male	13 (52)	17 (63)	33 (82)	21 (78)
Female	12 (48)	10 (37)	7 (18)	6 (22)
**Height (cm)**	1.67 ± 0.1	1.69 ± 0.1	1.76 ± 0.1	1.77 ± 0.1
**Mass (kg)**	64.6 ± 14.8	60.7 ± 8.0	76.3 ± 13.4	75.8 ± 11.6
**Injured leg, n (%)**				
Left	N/A	N/A	18 (45)	10 (37)
Right			22 (55)	17 (63)

Values are presented as mean ± standard deviation for continuous variables and as count (percentage) for categorical variables. Percentages are rounded to the nearest whole number. N/A = Not applicable.

### Equipment and calibration

The data were recorded using an Xsens Awinda system (Xsens Technologies B.V., Enschede, Netherlands), a wireless IMU-based motion capture system, using the manufacturer’s proprietary software (MVN Analyze, version 2023.0.0). Specifically, we recorded lower limb kinematics utilizing a model that consists of seven lightweight (16 grams) sensors, each containing a 3D accelerometer, a 3D gyroscope, and a 3D magnetometer.

Before data collection, participants’ anthropometric measurements were taken and entered into the MVN software to create a scaled personalized biomechanical model. Using velcro straps, the IMU sensors were then attached at the following standardized locations: on both feet, shanks, thighs, and the pelvis.

After the sensors’ placement, each participant performed a standard calibration routine, which consisted of the following steps:

- N-pose calibration: Participants stood in a neutral pose with feet parallel and shoulder-width apart, arms relaxed at the sides, and looking straight ahead. This establishes the default reference position for all segments.
- Walk calibration: Participants walked around the room, turned, and walked back to the starting position. This calibration helps establish the relationship between sensor orientations and anatomical axes, thereby determining walking direction.
- Calibration verification: The software provides feedback on the quality of alignment. The process was repeated if the calibration quality fell below the recommended threshold.

The recordings were captured at a frequency of 100 Hz and transmitted wirelessly to a laptop running Xsens MVN software, which implements proprietary sensor fusion algorithms to calculate joint kinematics.

### Research protocol

The walking protocol consisted of three tasks, each performed along a 20-meter corridor. In each task, participants walked in a straight line, without turning, at one of three self-selected speeds: normal, slow, and fast (faster than normal walking but not running). Standardized verbal cues given to participants, adapted from a prior study, were: “Walk across the walkway at a slow speed,” “Walk across the walkway at your normal speed,” and “Walk as fast as possible”^22^. For each speed condition, participants completed three consecutive trials. No external feedback was provided. Speeds were intentionally unconstrained to capture each participant’s natural pacing. Participants rested between trials and resumed when they confirmed readiness to proceed. Only the straight overground walking segments were used for analysis.

For participants in the ACL injury cohort, the initial assessment was conducted before their scheduled surgery, and the follow-up assessment was performed three months after surgery using identical procedures.

## Data Records

The dataset is publicly available on Zenodo under the Creative Commons Attribution 4.0 International (CC BY 4.0) license^23^. It is organized as four separate compressed folders, one for each cohort (Healthy Adults, Healthy Adolescents, ACLD, and ACLR). The dataset can be accessed at 10.5281/zenodo.15624356.

Values throughout the manuscript are presented as mean ± standard deviation or median [minimum-maximum].

The dataset is structured hierarchically to support group-level and longitudinal analyses. It includes four top-level subdirectories corresponding to participant groups:

- Healthy adults (HA): Adults without lower limb injury or known gait impairments
- Healthy adolescents (HK): Pediatric participants without known musculoskeletal conditions
- ACLD: ACL-injured participants recorded before surgery, during the ACL-deficient stage.
- ACLR: The same participants assessed three months post-ACL reconstruction (n = 27), a subset of the initial ACLD cohort (n = 40); 13 were lost to follow-up.
- Each group folder contains data from individual participants, labeled with unique identifiers (e.g., ACLD3, ACLR3, HA4, HK2). For ACL participants who completed both phases of data collection, their identifiers are consistent across time points (e.g., ACLD5 becomes ACLR5).

A summary of the folder hierarchy, file types, and contents is provided in Table 2.

**Table 2:** Data file structure and content summary.

Level	Folder/File	File Type	Contents	Notes
**Root**	COMPWALK-ACL/	Folder	All data	Includes four cohort folders and a metadata CSV file
**Metadata**	ID.csv	CSV	Participant-level metadata: ID, group, sex, age, height, mass, injured leg (if applicable)	Useful for filtering participants by cohort or demographics
**Cohort Level**	HA/, HK/, ACLD/, ACLR/	Folder	Participant data grouped by cohort (Healthy adults, Healthy adolescents, ACLD, ACLR)	ACLD and ACLR are longitudinal assessments of the same individuals (some missing follow-up)
**Participant Level**	HA01/, ACLD02/, etc.	Folder	Individual participant folder	Participant ID matches row in ID.csv
**Walking Speed Level**	slow/, normal/, fast/	Folder	Data for each of three walking speeds per participant	Each folder includes multiple trial files (e.g., normal-001)
**Trial Level**	normal-001.mvnx	MVNX	Raw time-series data, segment kinematics, sensor data, binary foot contacts	XML structured data for full biomechanical modeling
	normal-001.xlsx	XLSX	Flattened joint angles, center of mass, ergonomic kinematics	Tabular format for statistical analysis or spreadsheet-based analysis

HA = healthy adults; HK = healthy adolescents; ACLD = anterior-cruciate-ligament-deficient (pre-operative); ACLR = anterior-cruciate-ligament-reconstructed (≈3 months post-operative). MVNX = proprietary XML-structured motion file generated by Xsens MVN Analyze; XLSX = Microsoft Excel Open XML spreadsheet.

Within each participant’s folder, data are further subdivided into normal, slow, and fast walking speeds. Each speed folder contains multiple trials labeled sequentially (e.g., normal-001, slow-002, fast-003). Each trial consists of an .mvnx file paired with an .xlsx spreadsheet.

The .mvnx file provides a structured record of time-series data, including joint angles, segment orientations and positions, linear and angular velocities and accelerations, center of mass trajectories, and sensor-level data such as free acceleration, magnetic field, and orientation in both quaternion and Euler formats. The .mvnx file also includes binary foot contact information (for the heel and toe) and is not available in the .xlsx file.

The .xlsx file contains a flattened, tabular version of key time-series variables, organized into multiple labelled sheets. ‘Segment Orientation’, provided in both Quaternion and Euler angle formats for rotational data; ‘Segment Position’, detailing the 3D coordinates of each segment; and sheets for ‘Segment Velocity’, ‘Segment Acceleration’, ‘Segment Angular Velocity’, and ‘Segment Angular Acceleration’, which together provide a complete kinematic profile of the body’s movement.

The file also includes detailed information on joint angles. There are sheets for ‘Joint Angles’ and ‘Ergonomic Joint Angles’, with each type calculated using both ZXY and XZY rotational conventions. A key advantage of the .xlsx format, compared to the .mvnx file, is the use of clear anatomical labels for joint angle variables, such as “Left Ankle Dorsiflexion/Plantarflexion,” simplifying interpretation. Furthermore, the data includes a sheet for the ‘Center of Mass’, which provides the position, velocity, and acceleration of the body’s overall center of mass.

Finally, the workbook provides both raw and processed sensor outputs, including ‘Sensor Free Acceleration’, ‘Sensor Magnetic Field’ (a tri-axial magnetometer), and ‘Sensor Orientation’, with orientation available in both quaternion and Euler representations.

For analyses requiring steady-state walking, we trimmed the dataset during preprocessing by removing the first two and last two steps from each walking trial, thereby excluding the initial acceleration and final deceleration phases. While the full (untrimmed) dataset is provided with this manuscript, all group-level spatiotemporal comparisons and technical validation analyses were conducted using the trimmed data to ensure consistency and biomechanical relevance.

While both formats capture equivalent core kinematic and inertial data, the .mvnx file offers full fidelity for biomechanical modeling, whereas the .xlsx file provides greater readability and access for statistical analyses. Full details on the .mvnx structure and axis conventions are available in the Xsens MVN User Manual^24^.

At the root of the COMPWALK-ACL directory, a metadata file titled ID.csv provides participant information, including:

- ID: Unique participant code
- Group: Participant cohort (HA, HK, ACLD, ACLR)
- Sex: Male/Female
- Age: in years
- Mass: Body mass in kilograms
- Height: Stature in centimeters
- Injured leg: Side of injury (for ACL participants only)

The mean number of steps per participant is 182.9 ± 30.9 for all trials and 144.8 ± 29.5 for the trimmed dataset. The corresponding strides were 89.1 ± 15.4 and 70.0 ± 14.8, respectively. Table 3 shows the mean and median values of steps and strides per participant for each group, for both the untrimmed and trimmed datasets.

**Table 3:** Total steps and strides per participant by group (untrimmed and trimmed data).

Group	Steps (Mean ± SD)	Median [Range]	Strides (Mean ± SD)	Median [Range]
**All Trials**				
**Healthy Adults**	183.76 ± 31.74	181 [135–270]	89.28 ± 15.66	89 [65–132]
**Healthy Adolescents**	185.95 ± 17.21	183 [140–215]	89.82 ± 8.96	88 [67–105]
**ACLD**	189.40 ± 28.49	192 [120–254]	92.72 ± 14.17	93 [58–124]
**ACLR**	169.54 ± 39.18	172 [109–297]	82.73 ± 19.46	84 [52–146]
**Trimmed**				
**Healthy Adults**	146.16 ± 29.94	145 [99–234]	70.48 ± 14.79	71 [47–114]
**Healthy Adolescents**	130.68 ± 15.19	129 [96–159]	62.18 ± 8.0	61 [45–77]
**ACLD**	154.90 ± 27.88	159 [92–222]	75.47 ± 13.87	77 [44–108]
**ACLR**	139.85 ± 35.56	141 [85–257]	67.88 ± 17.66	68 [40–126]
**Overall**				
**All Participants (all trials)**	182.91 ± 30.91	182 [109–297]	89.10 ± 15.37	88 [52–146]
**All Participants (trimmed)**	144.79 ± 29.50	144 [85–257]	70.04 ± 14.81	69 [40–126]

Values are presented as mean ± standard deviation and median [minimum - maximum]. Trimmed refers to datasets with the first two and last two steps and strides of each walking trial removed to exclude acceleration and deceleration phases, keeping only the steady-state gait cycles. ACLD = Anterior cruciate ligament deficiency. ACLR = Anterior cruciate ligament reconstruction.

**Fig. 1:**
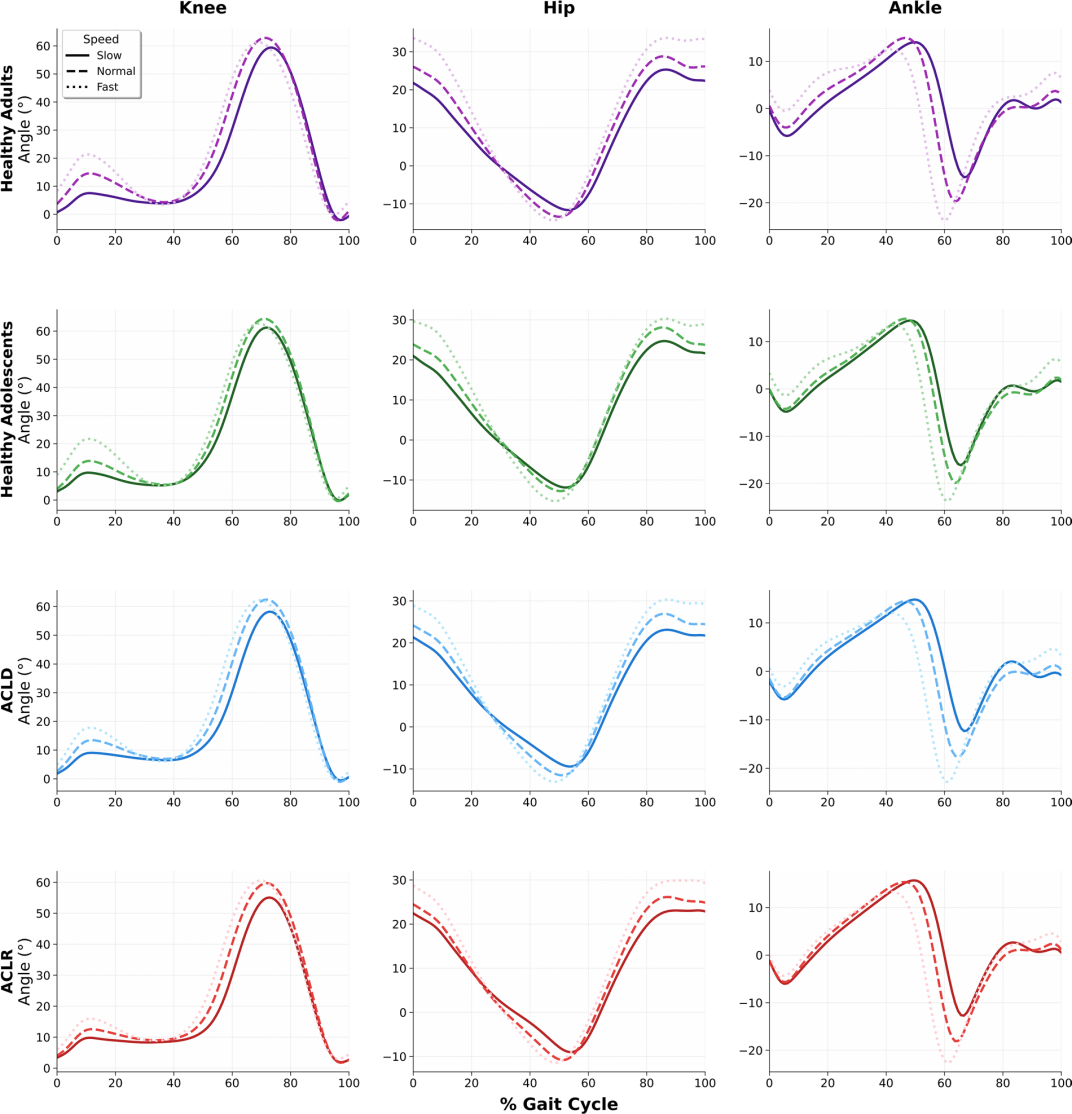
Sagittal-plane joint kinematics across walking speeds in healthy and injured cohorts. Averaged joint angle trajectories across all gait cycles from all trimmed walking trials of the healthy adult (n = 25), healthy adolescent (n = 27), ACLD (n = 40), and ACLR (n = 27) cohorts, with all trajectories time-normalized to 101 points. Solid, dashed, and dotted lines correspond to slow, normal, and fast walking trials, respectively. Positive values denote flexion (knee & hip) or dorsiflexion (ankle). Zero equals the anatomically neutral position. ACLD: Anterior Cruciate Ligament Deficiency; ACLR: Anterior Cruciate Ligament Reconstruction.

As an example of the joint-level kinematic data available in the dataset, Fig. 1 illustrates sagittal-plane trajectories of the hip, knee, and ankle joints across walking speeds in healthy adults. The data are averaged across all gait cycles from all trimmed trials in this cohort, highlighting speed-dependent changes in joint angles. This figure demonstrates how the .mvnx and .xlsx files can be used to extract joint-specific waveforms across conditions, supporting stride-level and time-normalized analyses across populations.

## Technical Validation

To assess if our IMU‐derived gait metrics are both reliable and physiologically meaningful across cohorts and speeds, we performed a series of statistical tests. All analyses in this section were conducted using the trimmed data, in which the first two and last two steps of each trial were excluded to focus on steady-state walking. This section describes the analyses performed to quantify within-participant consistency, speed effects, group differences, and concordance with manufacturer benchmarks.

Specifically, we evaluated (1) trial‐to‐trial consistency within each cohort, (2) responsiveness of key gait parameters to speed changes, (3) cohort‐specific differences, especially between the ACL‐injured group and healthy adults, and (4) comparisons with manufacturer-reported benchmarks from the Xsens whitepaper.

### Reliability (Within-Participant Consistency)

Within-participant reliability was assessed by calculating the coefficient of variation (CV) for five spatiotemporal gait parameters: gait speed, cadence, stride length, step width, and stride/step time. Across all groups and speed conditions, gait speed exhibited low intra-individual variability, with mean CV values ranging from 2.29% to 5.85%. Cadence showed mean CVs ranging from 2.00% to 5.02%, and stride length CVs ranged from 1.70% to 5.35%. Stride time and step time CVs ranged from 1.41% to 5.19% and 1.46% to 5.11%, respectively. In contrast, step width demonstrated the highest variability, with CVs ranging from 5.45% to 11.9%. Group-level mean values for these parameters across all cohorts are summarized in Table 4.

**Table 4:** Group-level spatiotemporal key parameters across cohorts.

Cohort	Gait Speed (meters/second)	Cadence (steps/minute)	Stride Length (meters)	Stride Time (seconds)
**Healthy Adults**	1.37 ± 0.47	112 ± 21.60	1.44 ± 0.28	1.12 ± 0.23
**Healthy Adolescents**	1.45 ± 0.40	113 ± 17.70	1.52 ± 0.25	1.09 ± 0.17
**ACLD**	1.24 ± 0.40	104 ± 18.30	1.39 ± 0.27	1.19 ± 0.21
**ACLR**	1.23 ± 0.38	102 ± 16.70	1.41 ± 0.24	1.21 ± 0.20

Values are presented as mean ± standard deviation. Gait speed, cadence, stride length, and stride time were averaged across all walking trials and speed conditions. ACLD = Anterior cruciate ligament deficiency. ACLR = Anterior cruciate ligament reconstruction.

### Speed Responsiveness

**Fig. 2:**
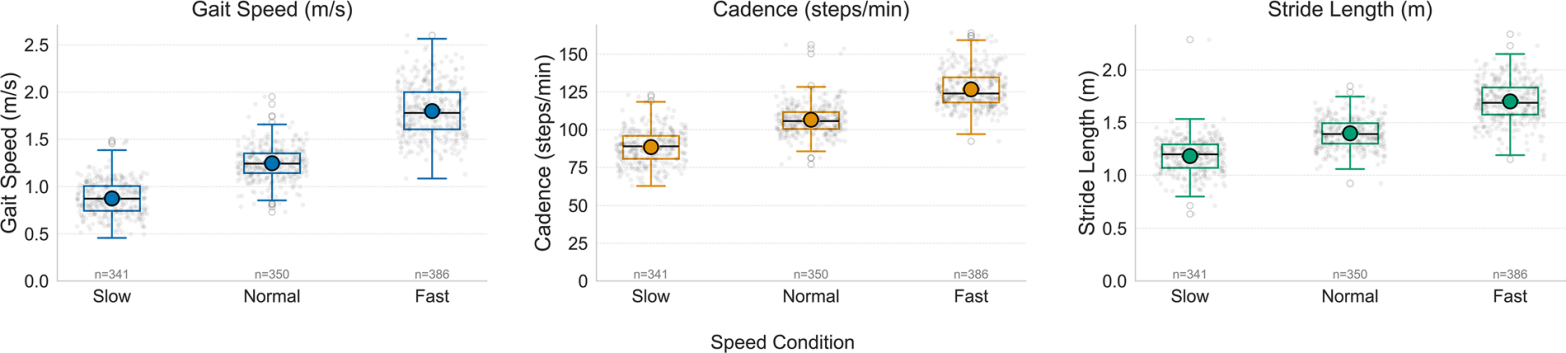
Responsiveness of gait metrics across speed conditions. The data presented pools together all participant cohorts (healthy adults, adolescents, ACLD, and ACLR) to illustrate the overall trend. Each grey point on the chart represents an individual walking trial. The data for each speed condition is summarized using a box plot, where the box shows the inter-quartile range, the horizontal line inside the box is the median, and the whiskers extend to 1.5 times the inter-quartile range. Gait speed is expressed in meters per second, cadence in steps per minute, and stride length in meters.

To validate the dataset’s ability to capture physiological responses, data from all participant cohorts (healthy adults, adolescents, ACLD, and ACLR) were pooled together to illustrate the overall trend and confirm the universal effect of speed cues. As seen in Fig. 2, gait speed, cadence, and stride length all increased significantly from slow to normal and fast walking speeds. Specifically, gait speed increased from 0.88 ± 0.01 m/s in the slow condition to 1.24 ± 0.01 m/s in the normal condition and 1.80 ± 0.01 m/s in the fast condition, with a significant main effect of speed (F(2, N) = 1736.99, p < 0.001). Cadence increased similarly, from 88.47 ± 0.59 steps/min (slow) to 106.59 ± 0.51 steps/min (normal) and 126.75 ± 0.61 steps/min (fast), with a significant main effect (F (2, N) = 1107.31, p < 0.001). Stride length also increased from 1.18 ± 0.01 m (slow) to 1.40 ± 0.01 m (normal) and 1.70 ± 0.01 m (fast), with a significant main effect of speed (F (2, N) = 918.62, p < 0.001). Post hoc analyses indicated that all pairwise comparisons between speed conditions were statistically significant (all p < 0.001). The speed-dependency in joint kinematics is further illustrated in Fig. 1, demonstrating the expected flexion-extension patterns across walking conditions.

Furthermore, the kinematic patterns observed in our IMU-derived dataset are consistent with those reported in the large-scale optical motion capture study by Schwartz *et al*. that examined gait across a range of walking speeds^25^. Despite differences in measurement technology and participant demographics, the fundamental speed-related effects on sagittal plane kinematics are consistent. In both our data (Fig. 1) and the reference study, increasing walking speed is associated with a greater range of motion at the hip, knee, and ankle.

### Cohort Comparisons by Speed

At all speed conditions, significant group differences were observed in gait speed, cadence, and stride length.

At slow walking speed, healthy adults walked significantly faster than the ACLD group (0.87 ± 0.02 m/s vs. 0.79 ± 0.01 m/s, p = 0.0037), with no other significant pairwise differences. Cadence was higher in healthy adults compared to both ACLD (88.94 ± 1.35 vs. 84.90 ± 0.93 steps/min, p = 0.0202) and ACLR (85.55 ± 1.04 steps/min, p = 0.1191), although only the ACLD comparison reached statistical significance. No significant pairwise differences were found in stride length at slow speed (p > 0.05 for all comparisons).

At normal walking speed, both ACLD and ACLR groups demonstrated significantly slower gait speed than healthy adults (ACLD: 1.19 ± 0.01 m/s; ACLR: 1.19 ± 0.02 m/s; healthy adults: 1.33 ± 0.02 m/s; both p < 0.001). Cadence was significantly lower in both ACL groups relative to healthy adults (ACLD: 104.33 ± 0.61; ACLR: 101.18 ± 1.05; healthy adults: 112.90 ± 1.26 steps/min; both p < 0.001), and the ACLR group had a marginally lower cadence than ACLD (p = 0.054). Stride length was significantly shorter in the ACLD group compared to healthy adults (1.37 ± 0.01 m vs. 1.42 ± 0.02 m, p = 0.0456).

At fast speed, both ACL groups exhibited significantly slower gait speed than healthy adults (ACLD: 1.73 ± 0.03 m/s; ACLR: 1.66 ± 0.03 m/s; healthy adults: 1.94 ± 0.03 m/s; both p < 0.001). Cadence was significantly lower in both ACL groups compared to healthy adults (ACLD: 123.56 ± 1.06; ACLR: 119.59 ± 1.34; healthy adults: 134.41 ± 1.22 steps/min; both p < 0.001), with a marginal difference between ACLD and ACLR (p = 0.0559). Stride length was significantly shorter in both ACL groups compared to healthy adults (ACLD: 1.67 ± 0.02 m, ACLR: 1.66 ± 0.02 m, healthy adults: 1.73 ± 0.02 m; p = 0.0351 and p = 0.0313, respectively).

Across speeds, healthy adults had higher gait speed, cadence, and longer stride length than those with ACLD/ACLR, particularly at normal and fast speeds. Differences between the ACLD and ACLR groups were minimal and did not reach statistical significance for any parameter across speed conditions. To specifically assess the dataset’s ability to capture differences due to ACL injuries, these analyses compared the adult clinical groups (ACLD/ACLR) only to their age-matched healthy adults. The adolescent cohort was excluded to isolate the effects of the injury from potential age-related confounding variables.

### Comparison with Xsens whitepaper

To assess the consistency of the dataset with manufacturer-reported values, we compared spatiotemporal gait parameters from our healthy adult cohort with the reported reference values in the Xsens MVN Analyze white paper^24^. At self-selected normal walking speed, the mean step length in our dataset was 70.5 cm, compared to 67.6 cm in the Xsens reference. The step width was 8.32 cm in our dataset, compared to 7.79 cm in the Xsens data. Similarly, the stride length in our dataset was 142 cm, compared to 134 cm in the whitepaper. Observed values were comparable in magnitude to those reported in the whitepaper.

## Usage Notes

The COMPWALK-ACL dataset cohort folders (ACLD, ACLR, healthy adults, and healthy adolescents) contain participant folders, each of which includes three speed-condition subfolders with paired trial files, .mvnx files, and .xlsx files.

The .mvnx files contain comprehensive time-series data of the raw sensor signals, segment orientations (quaternions), joint kinematics, and heel/toe contact events, suitable for advanced biomechanical or signal-processing analyses using custom scripts. The .xlsx files provide key joint angle and center of mass data in a tabular format and are easily importable into shared spreadsheets and statistical analysis software. Participants’ metadata are available in the ID.csv file and provide anonymised demographic metadata such as age, sex, height, mass, and injured side (for ACL participants).

The dataset supports a range of uses, including establishing normative joint-angle references across the adolescent–adult lifespan, benchmarking stride-segmentation or gait-speed classification algorithms, and tracking longitudinal changes after ACLR to evaluate recovery.

Potential users should note that the ACLD and ACLR groups comprise the same individuals. However, some participants were lost to follow-up, resulting in slightly different sample sizes between these two longitudinal measurement points.

Additionally, as with all IMU-based systems, users should be aware of limitation factors, such as environmental magnetic disturbances, that can impact data accuracy. However, the Xsens system uses a proprietary algorithm to mitigate such effects. Users should be further aware of several dataset-specific limitations. First, each overground trial spans only ~20 m, yielding 8–10 steady strides. Second, force-plate data and camera-based motion capture are not included. Therefore, without gold-standard measurements, the dataset cannot be used to validate any of the reported metrics, including joint kinematics and spatiotemporal parameters. Lastly, footwear and walkway surface were kept consistent within a session but not standardised across participants, possibly introducing between-subject differences.

## Data Availability

The dataset is available on Zenodo under the Creative Commons Attribution 4.0 International (CC BY 4.0) license and can be accessed at 10.5281/zenodo.15624356.

## Code availability

The full analysis pipeline used to extract spatiotemporal gait parameters from the Xsens MVNX files is openly available on GitHub (https://github.com/TomerIL/COMP-ACL/). This code includes functions for parsing MVNX files, detecting gait events, computing spatiotemporal parameters, and trimming trials to remove acceleration/deceleration phases. The repository also includes a few example scripts illustrating additional potential uses of the dataset.
